# Identification of Early-Stage Alzheimer's Disease Using Sulcal Morphology and Other Common Neuroimaging Indices

**DOI:** 10.1371/journal.pone.0170875

**Published:** 2017-01-27

**Authors:** Kunpeng Cai, Hong Xu, Hao Guan, Wanlin Zhu, Jiyang Jiang, Yue Cui, Jicong Zhang, Tao Liu, Wei Wen

**Affiliations:** 1 School of Computer Science and Engineering, Beihang University, Beijing, China; 2 International Research Institute for Multidisciplinary Science, Beihang University, Beijing, China; 3 School of Biological Science and Medical Engineering, Beihang University, Beijing, China; 4 Centre for Healthy Brain Ageing, School of Psychiatry, University of New South Wales, Sydney, NSW, Australia; 5 Neuropsychiatric Institute, Prince of Wales Hospital, Randwick, NSW, Australia; 6 Brainnetome Center, Institute of Automation, Chinese Academy of Sciences, Beijing, China; 7 National Laboratory of Pattern Recognition, Institute of Automation, Chinese Academy of Sciences, Beijing, China; 8 Beijing key laboratory of rehabilitation engineering for elderly, Beijing, China; Nathan S Kline Institute, UNITED STATES

## Abstract

Identifying Alzheimer’s disease (AD) at its early stage is of major interest in AD research. Previous studies have suggested that abnormalities in regional sulcal width and global sulcal index (g-SI) are characteristics of patients with early-stage AD. In this study, we investigated sulcal width and three other common neuroimaging morphological measures (cortical thickness, cortical volume, and subcortical volume) to identify early-stage AD. These measures were evaluated in 150 participants, including 75 normal controls (NC) and 75 patients with early-stage AD. The global sulcal index (g-SI) and the width of five individual sulci (the superior frontal, intra-parietal, superior temporal, central, and Sylvian fissure) were extracted from 3D T1-weighted images. The discriminative performances of the other three traditional neuroimaging morphological measures were also examined. Information Gain (IG) was used to select a subset of features to provide significant information for separating NC and early-stage AD subjects. Based on the four modalities of the individual measures, i.e., sulcal measures, cortical thickness, cortical volume, subcortical volume, and combinations of these individual measures, three types of classifiers (Naïve Bayes, Logistic Regression and Support Vector Machine) were applied to compare the classification performances. We observed that sulcal measures were either superior than or equal to the other measures used for classification. Specifically, the g-SI and the width of the Sylvian fissure were two of the most sensitive sulcal measures and could be useful neuroanatomical markers for detecting early-stage AD. There were no significant differences between the three classifiers that we tested when using the same neuroanatomical features.

## Introduction

Alzheimer's disease (AD) is the most common cause of dementia, with typical characteristics of progressive cognitive decline such as memory impairment and the degeneration of reasoning ability [1,2]. The onset of AD is insidious, and the decline in cognition may not manifest until effective interventions become difficult [1,3]. A previous study showed that facilitating intervention at an early stage could effectively alleviate the symptoms of the disease [4]. Therefore, early diagnosis of AD will benefit patients, families and society as a whole.

In the last twenty years, magnetic resonance imaging (MRI) has been widely used to study the neuroanatomical abnormalities of AD. MRI-based methods mainly examine brain volumes, focusing on certain particular regions of interest (ROIs) that are said to be adversely affected in the disease progression [5–7]. The commonly used methods include voxel-based morphometry (VBM) [8–10], which examines the whole brain on a voxel basis, and cortical thickness [11–13], which examines the vertices on the cortical surface to evaluate the thickness of the cortex of the whole brain or cortical ROIs. These methods investigate the atrophy manifested by AD pathology in the whole brain or regions of the brain.

Numerous neuroanatomical measures have been proposed for early AD detection [14]. However, early diagnosis of AD is still challenging [15–17]. Studies have found that sulcal changes are associated with normal aging [18,19] as well as cognitive decline [20]. Specifically, sulci in mild cognitive impairment (MCI) and AD brains were found to have less curvature, which sulcal widening observed decrease in normal controls; and less in depth, which sulcal depth observed reducing when comparing those of controls [20,21]. One of our own studies demonstrated that the global sulcal index decreased along with the increasing severity of AD, and the widths of nearly all individual sulci we investigated were larger in mild AD than in controls [22]. These results suggested that abnormalities in the global sulcal index and sulcal widths are potentially excellent features for the early diagnosis of patients with very mild AD. To our knowledge, very few studies have employed sulci for distinguishing between subjects in an early stage of AD and NC. Among the few studies that have used sulcal measures, Park and colleagues carried out a classification between AD and MCI using cortical thickness and sulcal depth [6,23]. They reduced the dimensions of feature space by applying principal component analysis (PCA), but this approach may come with two drawbacks: first, the principal components are linear combinations of all the original predictors rather than a representative subset of these predictors; the second is that most relevant components are selected regardless of the outcome of interest [24]. Recently, Hamelin and colleagues investigated the utility of sulcal width measurements in the diagnosis of Alzheimer’s disease. They demonstrated that sulcal width measurements were better markers than the hippocampal volume for identifying prodromal AD in patients aged younger than 65 years [25]. However, how well the sulci measures would contribute to the classification of the early-stage AD diagnosis with additional discriminative information was not clear. Furthermore, how each particular sulcus would assist in the detection of early-stage AD was even more difficult to determine.

Machine learning methods have recently become widely used for the classification of AD patients vs. normal controls by analyzing MRI scans [26–28]. Machine learning methods have shown potential for decoding high dimensional MRI data for discriminating AD from healthy brains and predicting the progression of the disease. Cui et al. used Support Vector Machine (SVM) as the classifier to predict the transition from normal cognition to mild cognitive impairment in two years and found that the prediction performance was better when using a combination of neuropsychological scores and morphological measures than using either of these two modalities alone [29]. Park and colleagues used SVM to separate normal controls from subjects with Alzheimer’s disease and mild cognitive impairment and achieved an accuracy of 94% [23]. Ota and colleagues used Logistic Regression to predict the progression to AD in amnestic mild cognitive impairment, and their findings suggested that the stratification of imaging biomarkers in amnestic MCI could be a good approach for improving prediction performance [30]. However, among neuroimaging studies of early-stage AD, there are very few studies that have compared the performance of different classification methods [31], and whether there are significant differences in performance between diverse classifiers trained on the same MRI dataset has yet to be explored.

In this study, we investigated the identification of early-stage AD using three different classifiers based on four modalities of imaging measures, including sulci, cortical thickness, cortical volume, and subcortical volume. The Mini-Mental State Examination (MMSE) [32] score and the composite of the four imaging measures were added to the classification. The Information Gain (IG) [33] method was used to perform feature selection, and the top 10 ranked feature subsets were generated to improve the accuracy of the classification. Specifically, we examined the performance of the global sulcal index and the index of the width of five individual sulci: the superior frontal, intra-parietal, superior temporal, central, and Sylvian fissure. The first aim of this study was to inspect the potential of sulci as discriminative biomarkers for the diagnosis of early-stage AD. The second was to examine if there were significant performance differences between various classification methods.

## Materials and Methods

### Ethics statement

For the purpose of this analysis, we used the OASIS database, which was previously collected under several study protocols at Washington University [34]. All studies were approved by the University’s Institutional Review Board (IRB). All subjects gave written informed consent at the time of study participation. The University’s IRB also provided explicit approval for open sharing of the anonymized data [34].

### Participants

Participants were drawn from the Open Access Series of Imaging Studies (OASIS) database (http://www.oasis-brains.org) [34]. Our investigation was restricted to right-handed participants who were at least 62 years old, the youngest age at which any individual in the OASIS database was classified as having early-stage AD. The individuals were classified on the basis of Clinical Dementia Rating scale (CDR) [35] scores as having normal cognition (CDR = 0, n = 75) or early-stage AD (CDR = 0.75 ± 0.25, n = 75). The age, sex, estimated total intracranial volume (eTIV), and Mini-Mental State Examination (MMSE) scores for the individuals in each of these groups were obtained directly from the OASIS database. Demographic characteristics of the participants are shown in Table 1.

**Table 1:** Participant demographic characteristics.

Characteristic	Normal (n = 75)	Early stage AD (n = 75)	*p*
Age (year)	75.41±7.829	76.24±7.573	0.512^a^
Sex (M/F)	29/46	20/55	0.117^b^
Education (year)	3.16±1.284	2.85±1.343	0.155^a^
MMSE	28.89±1.247	24.36±4.006	<0.05^a^

^a^Results of two-tailed t-test across two groups.

^b^Chi-square statistic.

### Image acquisition

For each participant, we obtained a single image with a high contrast-to-noise ratio from the OASIS database. As described by Marcus et al. [34], these images were produced by averaging across 3 or 4 motion-corrected T1-weighted magnetization-prepared rapid gradient-echo (MP-RAGE) images, which were acquired on a 1.5T Vision scanner (Siemens, Erlangen, Germany) within a single session during which cushioning and a thermoplastic face mask were used to minimize head movements. The MP-RAGE parameters were empirically optimized for gray-white contrast, with repetition time = 9.7 ms, echo time = 4.0 ms, inversion time = 20 ms, delay time = 200 ms, flip angle = 10°, orientation = sagittal, resolution = 256×256 matrix, slices = 128, and thickness = 1.25 mm with no gap, yielding 1 × 1 × 1.25 mm^3^ isotropic voxels.

### Image pre-processing

Cortical sulci were extracted from the images via the following three steps. First, we removed non-brain tissue to produce images containing gray matter (GM), white matter (WM) and cerebrospinal fluid (CSF) only. This was done by using the SPM5 skull-cleanup tool, warping a brain mask defined in the standard space back to the raw T1-weighted structural MRI scan. The brain mask was obtained from an automated skull stripping procedure based on the SPM5 [36]. Second, we segmented images into GM, WM and CSF using a fuzzy-classifier-based, anatomical segmentation method using BrainVisa (BV), after applying a field inhomogeneity bias correction [37]. Third, individual sulci were identified and extracted using the BV sulcal identification pipeline (http://brainvisa.info/). The medial surface of the cortical folds was calculated using a homotopic erosion technique [38], and a crevasse detector was used to reconstruct the sulcal structure as the medial surface from the two opposing gyral banks that spanned from the most internal point of the sulcal fold to the convex hull of the cortex [37]. A sulcal labeling tool incorporating 500 artificial neural network-based pattern classifiers [39] was used to label sulci. Sulci that were mislabeled by BV were manually corrected.

### Morphological measures

For each hemisphere, we calculated the global sulcal index (g-SI) as the ratio between the total sulcal area and outer cortical area [40]. We calculated the g-SI of each brain with no manual intervention using BV.

Also, for each hemisphere, we determined the average sulcal width for each of the five sulci: the superior frontal, intra-parietal, superior temporal, central, and Sylvian fissure. Sulcal width was defined as the average 3D distance between opposing gyral banks along the normal projections to the medial sulcal mesh [41,42]. The five sulci investigated in the present study were chosen because they are present in all individuals, large and relatively easy to identify after facilitating error detection and correction, and they are located on different cerebral lobes. We also calculated cortical thickness, cortical volume and subcortical volume. This was done using FreeSurfer (http://surfer.nmr.mgh.harvard.edu/) based on the atlas including 34 cortical regions of interest in each hemisphere [43].

### Feature selection

The purpose of feature selection was to improve classification performance by using an informative subset of features. The selected features should be AD specific. We selected features by using the Information Gain (IG) method, which selected features by considering the information gain value of each feature [33,44]. The information gain value is the change in the entropy of variable *X* from a prior state to a state that takes some information from the random variable *T*. Entropy of *X* is defined as follows:

$$H(X)=-\sum_{i=1}^{n}P(x_{i})\;\log_{2}\;P(x_{i})$$

where *x*_*i*_ is one of the classes, and n is the number of classes. The entropy of *X* after observing information from another variable *T* is defined:

$$H(X|T)=-\sum_{j}P(t_{j})\sum_{i}P(x_{i}|t_{j})\;\log_{2}\;P(x_{i}|t_{j})$$

where *P*(*x*_*i*_) is the prior probability for all values of *X*, and *P*(*x*_*i*_|*t*_*j*_) is the posterior probability of *X* given the values of *T*. The information gain can be calculated by the following formula:

IG(X,T)=H(X)−H(X|T)

According to this measure, given a dataset *X* and a feature *T*, *x*_*i*_ is the class of normal controls or the class of early-stage AD patients, and *P*(*x*_*i*_) is the probability of *X* belonging to class *x*_*i*_ [33,44]. For every feature of the dataset, the information gain was calculated. Feature selection was conducted in several steps. First, we calculated the information gain value of each feature. Second, a list of ranked features was generated based on the value of information gain in a descending order. Third, given a specified ranking number, all of the features with a ranking order that were higher than this number were selected to form a feature subset. The feature selection was performed on the training sets that were different combinations of the four measures (sulcal measures, cortical thickness, cortical volume and subcortical volume). For the purpose of reducing computational burden and avoiding over-fitting, we finally obtained a subset of top 10 ranked features for later classification.

### Classification and validation

**Fig 1:**
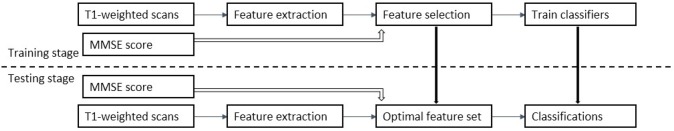
Overview of the classification procedure using T1-weighted scans and the MMSE score [32].

To validate whether different classifiers would result in significant differences, we evaluated the performances of three different classification algorithms including Naïve Bayes, Logistic Regression and SVM, which were available in the Waikato Environment for Knowledge Analysis (WEKA) package [45]. After an optimal feature subset was selected, Monte Carlo cross-validation was used to evaluate the methods [46]. Monte Carlo cross-validation (MCCV) works differently from K-folds cross-validation in that it generates more possible partitions which are done independently for each run. MCCV splits the dataset into training set and test set by sampling without replacement. In our experiment, the split ratio was set as 7:3, the 70% of the dataset were selected to form the training set. This process was then repeated 10 times. During each run, the classifier was trained with the training set and was then validated using the test set. For each classifier, the final classification results were the average of these 10 independent experiments. An overview of the classification procedure is shown in Fig 1.

The results of the different classifiers were compared using the performance metrics including accuracy, sensitivity and specificity. Furthermore, we plotted receiver operating characteristic (ROC) curves, and the areas under the ROC curves (AUC) were also calculated by averaging the trapezoidal approximations for the curve created by the True Positive Rate (TPR) and False Positive Rate (FPR).

## Results

For all three classifiers, performance characteristics were determined as the average of cross-validation experiments for each of the 8 approaches to classification: sulcal measures (SM) alone, cortical thickness (CT) alone, cortical volume (CV) alone, subcortical volume (SV) alone (Table 2), a combination of all of these measures (Table 3), a combination of all of these measures plus the MMSE scores (Table 4), and a combination of CT, CV, SV, and MMSE (Table 5), and, finally, a combination of SM, SV, and MMSE (Table 6).

**Table 2:** Classification results using sulcal measures, cortical thickness, cortical volume and subcortical volume separately.

Feature Set	Classifier	Accuracy	Sensitivity	Specificity	AUC
Sulcal Measures	Naïve Bayes^a^	0.682	0.667	0.659	0.663
	Logistic Regression^b^	0.736	0.733	0.735	0.761
	SVM^c^	0.712	0.711	0.712	0.743
Cortical Volume	Naïve Bayes	0.714	0.711	0.708	0.781
	Logistic Regression	0.733	0.733	0.732	0.82
	SVM	0.756	0.756	0.754	0.755
Cortical Thickness	Naïve Bayes^a^	0.759	0.756	0.752	0.812
	Logistic Regression^b^	0.733	0.733	0.732	0.775
	SVM^c^	0.735	0.733	0.731	0.732
Subcortical Volume	Naïve Bayes	0.732	0.711	0.704	0.751
	Logistic Regression	0.78	0.778	0.776	0.858
	SVM	0.768	0.756	0.75	0.753

^a b c^ There were no significant difference between the Sulcal Measures and Cortical Thickness using two-tailed t-test (p > 0.3).

**Table 3:** Classification results using combined data including sulcal measures, cortical thickness, cortical volume and subcortical volume.

Feature Set	Selected Attributes	Classifier	Accuracy	Sensitivity	Specificity	AUC
SM,CT,CV,SV	Hippocampus L	Naïve Bayes	0.832	0.822	0.818	0.874
	Hippocampus R					
	Amygdala R					
	Parahippocampal_volume R	Logistic Regression	0.815	0.8	0.795	0.866
	Precuneus_volume L					
	Precuneus_thickness R					
	Supramarginal_thickness R					
	g-SI L	SVM	0.846	0.822	0.816	0.88
	Entorhinal_volume R					
	Superiorparietal_volume R					

Abbreviations: SM-- sulcal measures; CT-- cortical thickness; CV-- cortical volume; SV-- subcortical volume

L-- Left hemisphere; R-- Right hemisphere.

**Table 4:** Classification results using combined data including sulcal measures, cortical thickness, cortical volume, subcortical volume and the MMSE score.

Feature Set	Selected Attributes	Classifier	Accuracy	Sensitivity	Specificity	AUC
SM,CT,CV,SV,MMSE	MMSE	Naïve Bayes^a^	0.894	0.867	0.861	0.893
	Hippocampus L					
	Hippocampus R					
	Amygdala R	Logistic Regression^a^	0.878	0.867	0.863	0.864
	Parahippocampal_volume R					
	Precuneus_volume L					
	Precuneus_thickness R	SVM^a^	0.909	0.889	0.884	0.826
	Supramarginal_thickness R					
	g-SI L					
	Entorhinal_volume R					

Abbreviations: SM-- sulcal measures; CT-- cortical thickness; CV-- cortical volume; SV-- subcortical volume

L-- Left hemisphere; R-- Right hemisphere

^a^ Significantly different from single measure using a two-tailed t-test (p<0.001).

**Table 5:** Classification results using cortical thickness, cortical volume, subcortical volume and the MMSE score.

Feature Set	Selected Attributes	Classifier	Accuracy	Sensitivity	Specificity	AUC
CT,CV,SV,MMSE	MMSE	Naïve Bayes	0.845	0.844	0.843	0.9
	Hippocampus L					
	Hippocampus R					
	Amygdala R	Logistic Regression	0.832	0.822	0.818	0.838
	Parahippocampal_volume R					
	Precuneus_volume L					
	Precuneus_thickness R	SVM	0.85	0.844	0.841	0.879
	Supramarginal_thickness R					
	Entorhinal_volume R					
	Superiorparietal_volume R					

Abbreviations: CT-- cortical thickness; CV-- cortical volume; SV-- subcortical volume.

L-- Left hemisphere; R-- Right hemisphere.

**Table 6:** Classification results using sulcal measures, subcortical volume and the MMSE score.

Feature Set	Selected Attributes	Classifier	Accuracy	Sensitivity	Specificity	AUC
SM,SV,MMSE	MMSE	Naïve Bayes	0.869	0.867	0.865	0.899
	Hippocampus L					
	Hippocampus R					
	Amygdala R	Logistic Regression	0.862	0.844	0.839	0.858
	g-SI L					
	g-SI R					
	Amygdala L	SVM	0.862	0.844	0.839	0.885
	Accumbens L					
	Sylvian L					
	Sylvian R					

Abbreviations: SM-- sulcal measures; SV-- subcortical volume.

L-- Left hemisphere; R-- Right hemisphere.

### Performances of using different measures

The results of the classifications using sulcal measures (sulcal width and global sulcal index), cortical thickness, cortical volume and subcortical volume separately are shown in Table 2. The table shows that classification using sulcal measures, cortical thickness and cortical volume achieved similar performances (p > 0.3). From the perspective of single modality measurements, using the sulcal measures, the accuracies of the classifiers ranged from 68.2% to 73.6% (sensitivity ranged from 66.7% to 73.3% and specificity 65.9% to 73.5%) and were similar to the results using cortical thickness (accuracy was from 73.3% to 75.9%, sensitivity from 73.3% to 75.6%, specificity from 73.1% to 75.2%) or cortical volume (accuracy ranged from 71.4% to 75.6%, sensitivity 71.1% to 75.6%, and specificity 70.8% to 75.4%). Subcortical volume outperformed all of the other three measures (p < 0.05), achieving accuracies of three classifiers ranging from 73% to 78% (sensitivity ranged from 71.1% to 77.8% and specificity 70.4% to 77.6%). Compared with cortical thickness and cortical volume, sulcal measures achieved comparable performances with a relatively lower dimension feature (12 dimensions) vector. This is important since fewer representative features help better understand the biological basis and the progression of cognitive disorders, and reduce the risk of overfitting. Table 3 to Table 6 show the classification outcomes of the combinations of the four neuroimaging measures and the MMSE scores. When using all of the imaging features, we obtained accuracies that ranged from 81.5% to 84.6%, and the highest AUC was generated by the SVM classifier as shown in Table 3. Additionally, classification using all of the imaging features plus the MMSE scores improved the accuracy to almost 91% with an AUC score of 0.89 (Table 4). If we remove sulcal measures from the classification, then the accuracies were reduced from 87.8%-90.9% to 83.2%-85% as shown in Tables 4 and 5. Finally, Table 6 shows the results of combining sulcal measures, subcortical volume and the MMSE scores as the input to test the effectiveness of sulcal measures by replacing thickness and volume; the best accuracy was 86.9%, and the highest AUC was 0.899. Comparing Tables 5 and 6, we showed that those two combinations achieved accuracies ranging from 83% to 86%, with the specificities ranging from 81.8% to 86.5%, sensitivities from 82.2% to 86.7% and AUCs from 0.838 to 0.9. The performance of classifiers was improved by combining different measurement modalities (p < 0.001).

### Performances of different classifiers

**Fig 2:**
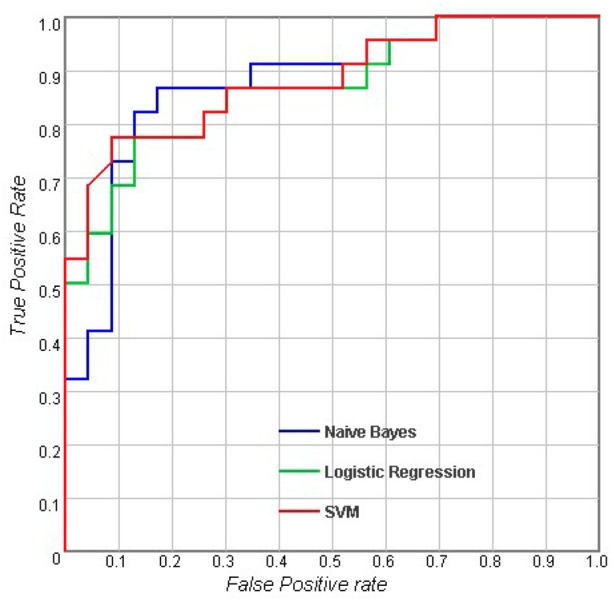
Receiver operating characteristic (ROC) curves for using combined features including sulcal measures, cortical thickness, cortical volume and subcortical volume.

**Fig 3:**
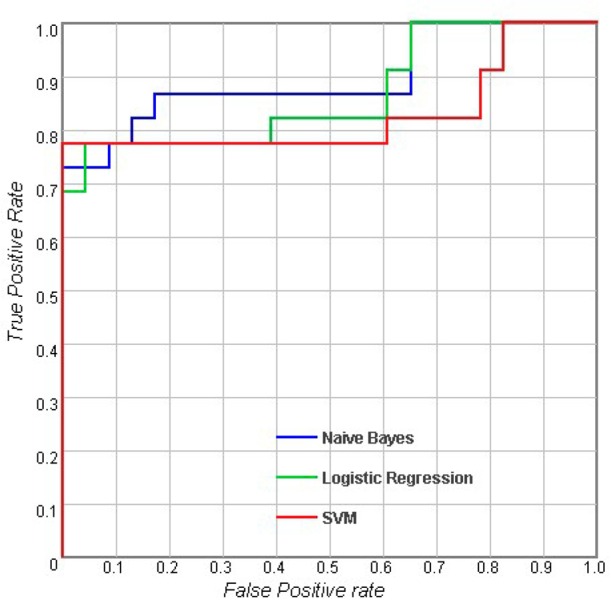
Receiver operating characteristic (ROC) curves for using combined features including sulcal measures, cortical thickness, cortical volume, subcortical volume and the MMSE score [32].

**Fig 4:**
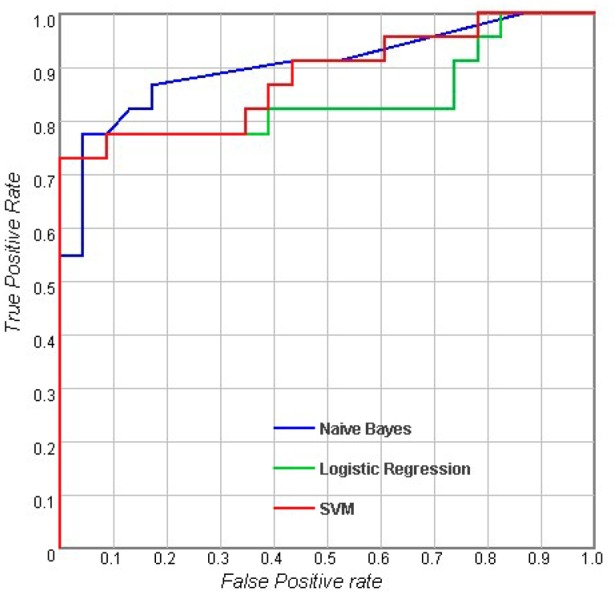
Receiver operating characteristic (ROC) curves for using combined features including cortical thickness, cortical volume, subcortical volume and the MMSE score [32].

**Fig 5:**
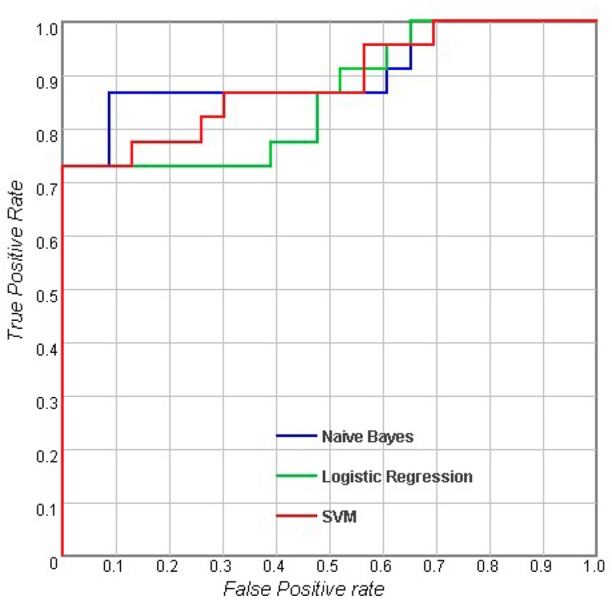
Receiver operating characteristic (ROC) curves for using combined features including sulcal measures, subcortical volume and the MMSE score [32].

Table 2 shows the results of the classifications using measurements of a single type. For the sulcal measures, Logistic Regression (LR) outperformed Naive Bayes and SVM with an accuracy of 73.6%, and the AUC of LR was also higher than the AUCs of the other classifiers. When considering cortical volume and cortical thickness, there were no obvious differences between the different classifiers. For subcortical measures, LR performed better than the others with an accuracy of 78% and an AUC of 0.858. In general, the results showed that with the same measurement modality, there were no significant differences between the performances of these three classifiers (p > 0.05). Figs 2–5 show the corresponding ROC curves of the three classifiers based on the four combinations of measures. According to the classification results and ROC curves, there were no significant differences in the classification performances of the three classifiers given the same feature set (p > 0.05).

### Discriminating features

The Information Gain method was used to select a subset of features that were most discriminative between subjects with early-stage AD and NC. For each combined feature set, we listed the top 10 selected features in Table 3 to Table 6. All four measures, including sulcal measures, cortical thickness, cortical volume and subcortical volume, were included in the subset. The hippocampus, amygdala, parahippocampus, precuneus, g-SI, supramarginal cortex and entorhinal cortex showed higher discriminability than other features. Table 4 shows that when adding the MMSE scores, the performance improved further. MMSE was also selected by the Information Gain method. Considering the results in Table 3 to Table 6, the MMSE scores, hippocampus, amygdala, parahippocampus, precuneus, supramarginal cortex, g-SI and Sylvian fissure were much more sensitive than the rest of the features.

## Discussion

The present study investigated the use of sulcal measures in the diagnosis of early-stage AD, and we evaluated the performance of three different classification methods. In addition, we selected the most discriminative features that would most contribute to the diagnosis of early-stage AD by using IG algorithm.

Many previous studies using imaging biomarkers for AD classification computed cortical thickness and volume as predictors. Eskildsen et al. combined the measurements of structural pathologic patterns, measured by analyzing morphologic alterations, and cortical thickness measures to predict AD among subjects with mild cognitive impairment from a single T1-weighted MRI scan. They reported the accuracy of 72% using a simple linear discriminant classifier [47]. Savio et al. applied four different models of artificial neural networks (Backpropagation, Radial Basis Networks, Learning Vector Quantization Networks and Probabilistic Neural Networks) to identify patients with early-stage AD by using gray matter volume as imaging feature. They achieved an accuracy ranging from 66% to 83% [48]. However, their study included only 98 females, which made their study not representative. By contrast, our study had a balanced population that included 150 participants with 75 early-stage AD patients (55 female subjects) and 75 normal controls (46 female subjects). We compared using cortical thickness and cortical volume separately to identify early-stage AD, and achieved accuracies close to 76% for both. Some previous studies explored the performance when adding sulcal measures. Park et al. trained SVM with cortical thickness, sulcal depth and their combinations to differentiate AD patients and normal controls [6]. Furthermore, they tested the performance of using combined features with ROI by eliminating non-informative vertex values that were not from ROIs. Based on the data of 25 AD patients and 50 NC, they reported 85% accuracy achieved by using the combination of features, and improved the accuracy to 90% accuracy by using the combination of features with the ROIs. As a comparison, we combined sulcal width and three other common neuroimaging morphological measures (cortical thickness, cortical volume, and subcortical volume) to differentiate early-stage AD patients and normal controls, and achieved an accuracy of 90.9%. Our results showed that adding sulcal measures could improve the classification performance.

Our study confirmed that sulcal measures were discriminative between subjects with early-stage AD and NC. The g-SI and Sylvian fissure were the most sensitive among all of the sulcal measures, which indicated that they could be useful neuroanatomical markers for early-stage AD. This was consistent with a previous report [22]. Notably, sulcal features and cortical features may reflect brain atrophy in similar aspects. When comparing classification performances that use sulcal features and cortical features separately, there was no significant difference between them (see Table 2). When combining sulcal features and cortical features as the original predictors for feature selection, fewer sulcal features were selected. The effectiveness of the sulcal features might be underestimated when combining sulcal features and cortical features because sulcal widening could be associated with cortical thinning at the same time [42,49]. However, it is interesting that as shown in Tables 4 and 6, the g-SI was always selected when using two different combinations of feature sets. The g-SI appeared to be a superior imaging feature in the classification because it reflected a global change in the human cortex. In addition to the g-SI, we noticed that the Sylvian fissure was also more sensitive in separating subjects with early-stage AD from NC than the other four individual sulci.

Using the same modalities or combinations of features, the three different classifiers achieved classifications close in accuracy, sensitivity, specificity and AUC, suggesting that neuroimaging features may play a more important role than classifiers of different algorithms in the diagnosis of early-stage AD. However, the accuracy, sensitivity and specificity of the classification were not satisfactory for clinical use, and future research should focus on the selection of sensitive imaging, cognition, and clinical features as well as features in other domains to improve the classification performance in order for this technique to eventually be used for clinical applications.

It is worth noting that the hippocampus, amygdala, parahippocampus, precuneus, supramarginal cortex, entorhinal cortex, g-SI and Sylvian fissure showed significant differences between normal controls and early-stage AD patients. The hippocampus plays an important role in memory and spatial navigation [50–52]. Atrophy of the hippocampus is considered an important biomarker of memory impairment and is related to early-stage AD [53,54]. The amygdala is considered to be related to the processing of memory and emotional function [55,56]. Amygdala atrophy can also be a characteristic of AD [57,58]. Previous reports have suggested that individuals with early-stage AD have lower levels of global cortex gyrification, which were measured by the g-SI [22]. In addition, previous reports have shown that among all the sulci, the Sylvian fissure exhibits the largest increase in width between normal cognition individuals and mild AD patients [22]. According to these findings, the features that were selected using the Information Gain method can be explained by the biological and pathological changes in AD.

Sylvian fissure is the sulcus that separates the frontal and temporal lobes. Post-mortem studies reported that AD-associated widening specifically of the superior frontal and superior temporal sulci [59,60]. Furthermore, one of our own earlier studies found that Sylvian fissure exhibited the largest increase in width between individuals with normal cognition and those with mild AD [22]. As the most discriminating feature in our current study, we found that Sylvian fissure could be a useful neuroanatomical marker of early-stage AD.

A limitation of our study is that the individuals were classified using CDR scale. While the CDR is a scale used to characterize six domains of cognitive and functional performance applicable to Alzheimer disease and related dementias, it is not specific for characterizing dementia of the Alzheimer’s type (DAT) [61]. The OASIS dataset we analyzed had a global CDR of 0 for cognitively normal, and CDRs of 0.5 and 1 for very mild, and mild dementia respectively. There was no DAT with OASIS.

In conclusion, our experiments showed that sulcal measures could be useful neuroanatomical markers in differentiating early-stage AD and normal controls. The g-SI and Sylvian fissure were the most significant features among the sulcal measures that we employed. Although sulcal measures did not perform better than other commonly used neuroimaging indices, the results indicated that adding sulcal measures could improve the classification performance. Our study also demonstrated that there were no significant differences in the final classification between the three different classifiers. Among all of the features investigated, the hippocampus, amygdala, parahippocampus, precuneus, supramarginal cortex, entorhinal cortex, g-SI and Sylvian fissure width were the most sensitive for early-stage AD detection. These features could therefore help understand the pathology of this condition in clinical applications.
